# Effects of Varying Pulse Width and Frequency of Wireless Stimulation in Rat Sciatic Nerve

**DOI:** 10.1109/EMBC46164.2021.9631070

**Published:** 2021-11

**Authors:** Rebecca A. Frederick, Philip R. Troyk, Stuart F. Cogan

**Affiliations:** Bioengineering Department at The University of Texas at Dallas, 800 W. Campbell Rd., BSB 11, Richardson, TX 75080 USA.; Biomedical Engineering Department at the Illinois Institute of Technology, Chicago, IL 60616 USA. He is also with Sigenics, Inc., Chicago, IL 60616 USA.; Bioengineering Department at The University of Texas at Dallas, 800 W. Campbell Rd., BSB 11, Richardson, TX 75080 USA.

## Abstract

Peripheral nerve stimulation is a commonly used method for assisting movements after spinal cord injury, stroke, traumatic brain injury, and other types of neurological damage or dysfunction. There are many different patterns of electrical stimulation used to accomplish movement. And so, our study investigated stimulation with a wireless floating microelectrode array (WFMA) in comparison to previously reported data on functional electrical stimulation. To determine the effect on hindlimb movement, we tested a range of frequencies and pulse widths using WFMAs that were implanted in the rat sciatic nerve for 38 weeks. Frequencies between 1 and 50 Hz did not change the minimum current amplitude required to elicit movement in the hindlimb. Increasing pulse width from 57.2 to 400.4 μs decreased the minimum current required but had an associated increase in total charge applied per pulse. Overall, the WFMA provides a stable wireless peripheral nerve interface suitable for functional electrical stimulation.

## I. Introduction

Functional electrical stimulation (FES) is often used to control movements in the upper and lower limbs after a patient suffers a neurological injury or experiences dysfunction of the neuromuscular system. Devices implanted on peripheral nerves are becoming more common. There is ongoing research on high-frequency burst patterns, effect of interphase delay, and how to vary stimulation parameters to minimize muscle fatigue or to activate central mechanisms in the brain and spinal cord. Yet, there is no set standard for stimulation patterns used to generate movement. Most modern devices utilize biphasic constant-current stimulation with frequencies between 20 and 50 Hz [1] and pulse widths of 50 to 500 μs [1].

The purpose of this work was to identify the effects of varying pulse width and frequency of electrical stimulation on hindlimb movements produced by an intraneural electrode array with a wireless interface. We sought to determine the frequency at which movements in the hindlimb transition from distinguishable twitches (corresponding to each individual electrical pulse) to single movements lasting for the duration of the applied stimulation train. We also sought to determine the effect of changing frequency and pulse width on the minimum current amplitude required to evoke movement in the hindlimb.

## II. Methods

**Figure 1.**
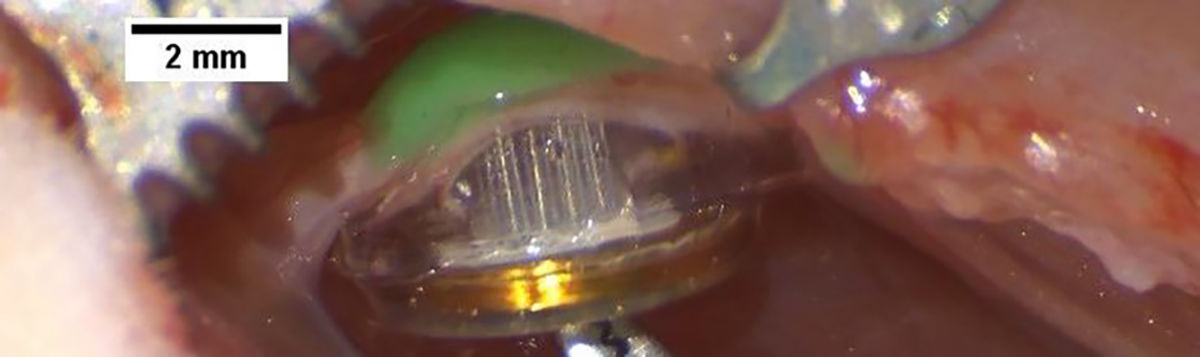
Wireless floating microelectrode array (WFMA) after implantation in the rat sciatic nerve. Device is secured with a silicone epoxy (green).

All animal procedures were performed in accordance with the guidelines of the Institutional Animal Care and Use Committee of The University of Texas at Dallas. Wireless floating microelectrode arrays (WFMAs) [2–4] with sixteen 2000 μm^2^ activated iridium oxide film (AIROF) stimulation electrodes were implanted in the left sciatic nerve of n=6 female Sprague Dawley rats (Figure 1). A skin incision was made parallel to the femur, and the biceps femoris muscle was retracted to reveal the sciatic nerve. Connective tissue around the entire circumference of the nerve was separated, without removing the epineurium, so that the nerve could be manipulated for device implantation. The sciatic nerve was gently lifted with a custom-made glass hook, and the WFMA was placed underneath such that electrodes pointed towards the nerve and the flat coil surface rested on the underlying muscle tissue. The nerve was lowered into a silicone nerve guide channel and the nerve and device carefully pressed together until electrodes penetrated the sciatic nerve. The WFMA was secured with silicone epoxy (Kwik-Cast™) and device communication and electrode state were confirmed by using reverse telemetry to record the voltage transient response to constant-current pulses. The muscle was then closed using 4–0 silk suture and the skin was closed with 11 mm staples. Animals were given slow-release buprenorphine immediately after surgery and at 72 hr post-op to manage pain. Animals were given cefazolin immediately after surgery and water with sulfamethoxazole for one week post-op as a prophylactic.

Stimulation trials were performed under isoflurane anesthesia (1.0 – 2.0 %) to determine the threshold current required to generate visible movement in the hindlimb. Trials were completed once per week starting on post-op week 1 (day 9), then once every two weeks starting on post-op week 8 and continuing through the final session on week 38. On week 38, additional stimulation trials were performed to determine the effect of pulse width and frequency on the current threshold for motor recruitment. Stimulation was applied at 1 Hz with a 200.2 μs pulse width to determine the baseline current threshold (I_th_) for each electrode individually. We then determined I_th_ for stimulation with 200.2 μs pulse width at 2, 5, 10, 20, 30, 40, and 50 Hz frequencies. Finally, we determined I_th_ for stimulation at 1 Hz with 57.2, 114.4, 314.6, and 400.4 μs pulse widths. I_th_ values as a function of pulse width (PW) were used to calculate the charge required to generate visible motion in the limb (Q_th_ = I_th_ × PW).

## III. Results

We implanted 96 electrodes in total in the sciatic nerve of n=6 rats. Here, we report the threshold values for each electrode after WFMA devices had been implanted in the animals for 38 weeks. At week 38, 17 electrodes did not evoke movement in the left hindlimb when tested up to the maximum current output of the WMFA. And so, no data is reported for 17 electrodes: Animal 01 - E01, E03, E04, E10, E14, E15, E16; Animal 02 - E02; Animal 03 - E14, E15; Animal 04 - E02, E03; Animal 05 - E14, E15; Animal 06 - E01, E02, E04. Additionally, 11 electrodes had baseline I_th_ values that were too high to investigate pulse widths shorter than the baseline measurement (particularly 57.2 μs) that would require higher I_th_ values potentially exceeding polarization limits known to lead to damage of the electrode and surrounding tissue. Therefore, no data was recorded for frequency or pulse width modulation of 11 electrodes that did cause movement in the hindlimb on week 38: Animal 01 - E02, E07; Animal 02 - E04, E12, E16; Animal 03 - E16; Animal 04 - E01, E04, E14, E16; Animal 06 - E03.

### A. Baseline Current Thresholds

**Figure 2.**
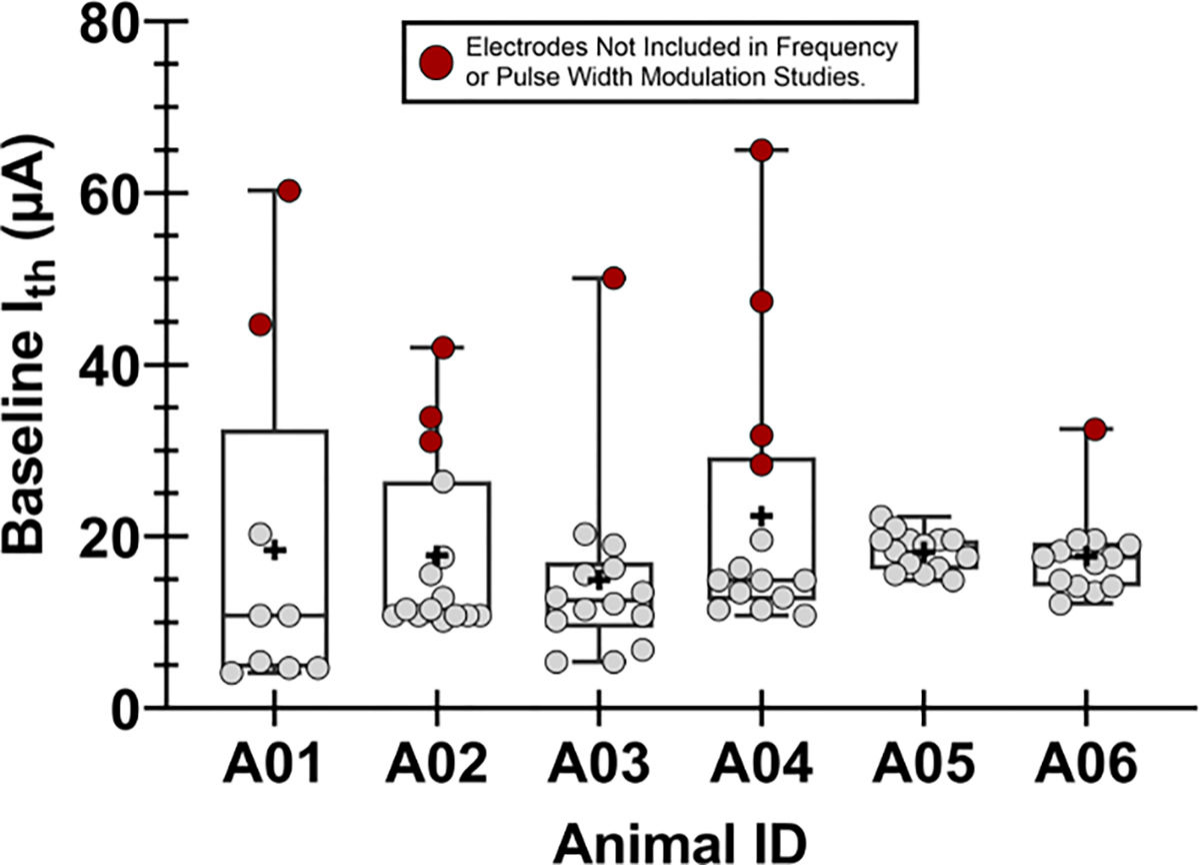
Baseline current thresholds for motor recruitment (I_th_) for each animal in the study. Stimulation frequency was at 1 Hz and pulse width was set to 200.2 μs. All data were recorded during the final stimulation testing session on post-implantation week 38. For each animal, the mean value of all electrodes evoking movement is indicated by a “+” symbol.

Motor recruitment thresholds recorded on postimplantation week 38 with stimulation at 1 Hz and 200.2 μs pulse width ranged from 4.1 to 38.6 μA across all electrodes and all animals. The average and standard deviation for baseline I_th_ was 14.2 ± 6.5 μA. Different movements were evoked at I_th_ for different electrodes, as the spatial arrangement of the WFMA layout resulted in electrodes inserting into different fascicles within the sciatic nerve. Most electrodes evoked plantar flexion at the ankle, while other electrodes evoked dorsiflexion at the ankle, toe flexion or extension, or movement of a single toe. Figure 2 shows the baseline I_th_ value for each electrode where the mean value is indicated by a “+” symbol and electrodes that were excluded from further studies of frequency and pulse width modulation are shown in red.

### B. Effects of Varying Frequency

**Figure 3.**
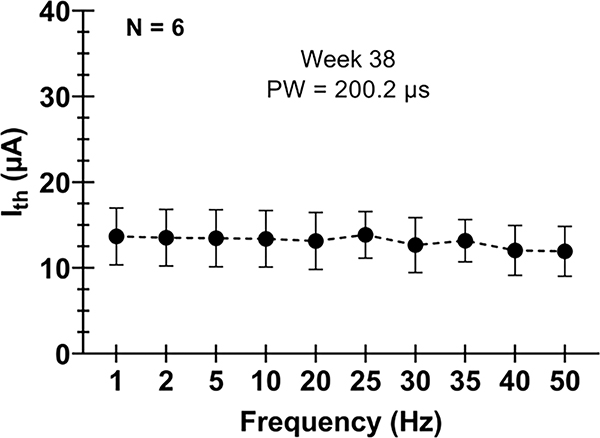
Current thresholds for motor recruitment (I_th_) as a function of frequency (pulse width set to 200.2 μs). Error bars represent standard deviation across six animal subjects implanted with a WFMA.

We found that changing the frequency of the applied stimulation (with 200.2 μs pulse width) had no effect on the I_th_ value for the range tested here (1 to 50 Hz). At most, the difference in Ith between 50 Hz stimulus and 1 Hz stimulus was 6.8 μA and the average difference between 50 Hz and 1 Hz I_th_ values was 1.8 ± 1.6 μA. Figure 3 shows average I_th_ values as a function of stimulation frequency.

Changing stimulation frequency did, however, drastically change the type of movement we observed. Low frequency stimulation (1 Hz to 20 Hz) elicited single movements of the hindlimb at the same rate as the applied pulses (1 per second, 1 per 0.5 seconds, etc.). Higher frequency stimulation (30 to 50 Hz) gradually elicited a tonic muscle contraction that was maintained from the start of the applied stimulus until stimulation was stopped.

### C. Effects of Varying Pulse Width

**Figure 4.**
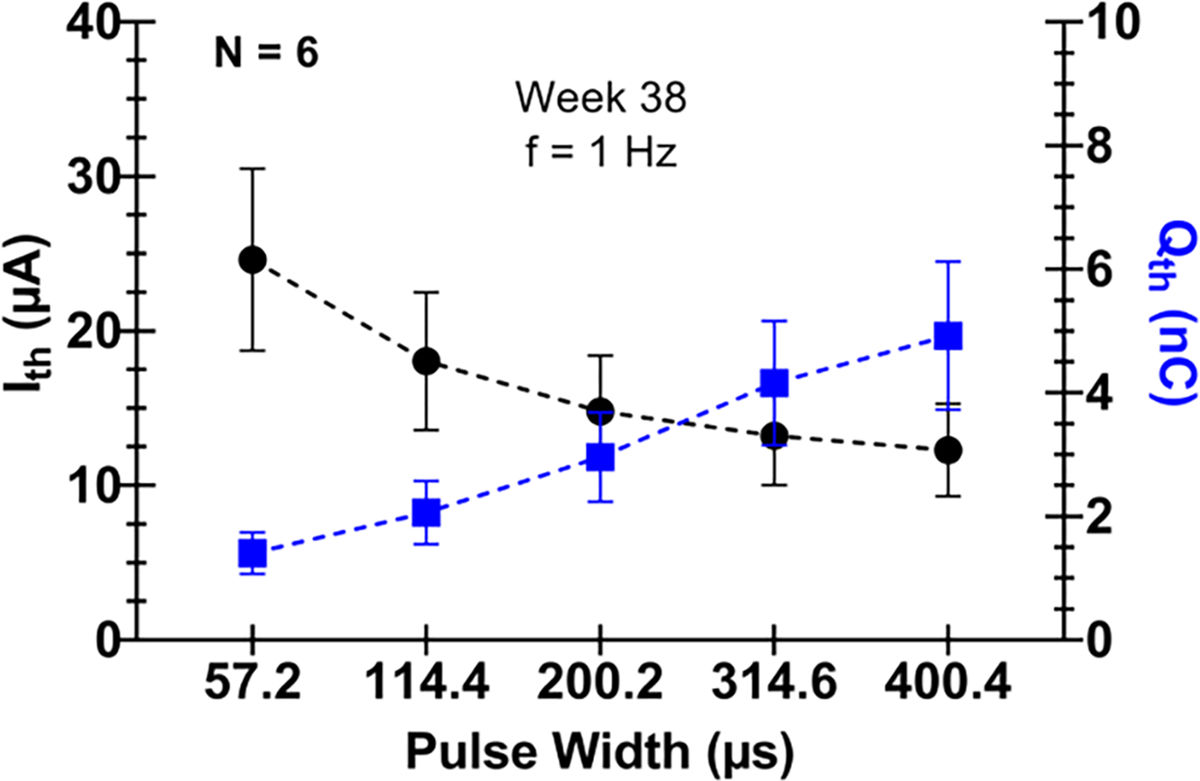
Current thresholds for motor recruitment (I_th_) as a function of pulse width (frequency set to 1 Hz) plotted on left y-axis. Charge per pulse required for motor recruitment (Q_th_), calculated from I_th_ values, plotted on right y-axis. Error bars represent standard deviation across six animal subjects implanted with a WFMA.

**Figure 5.**
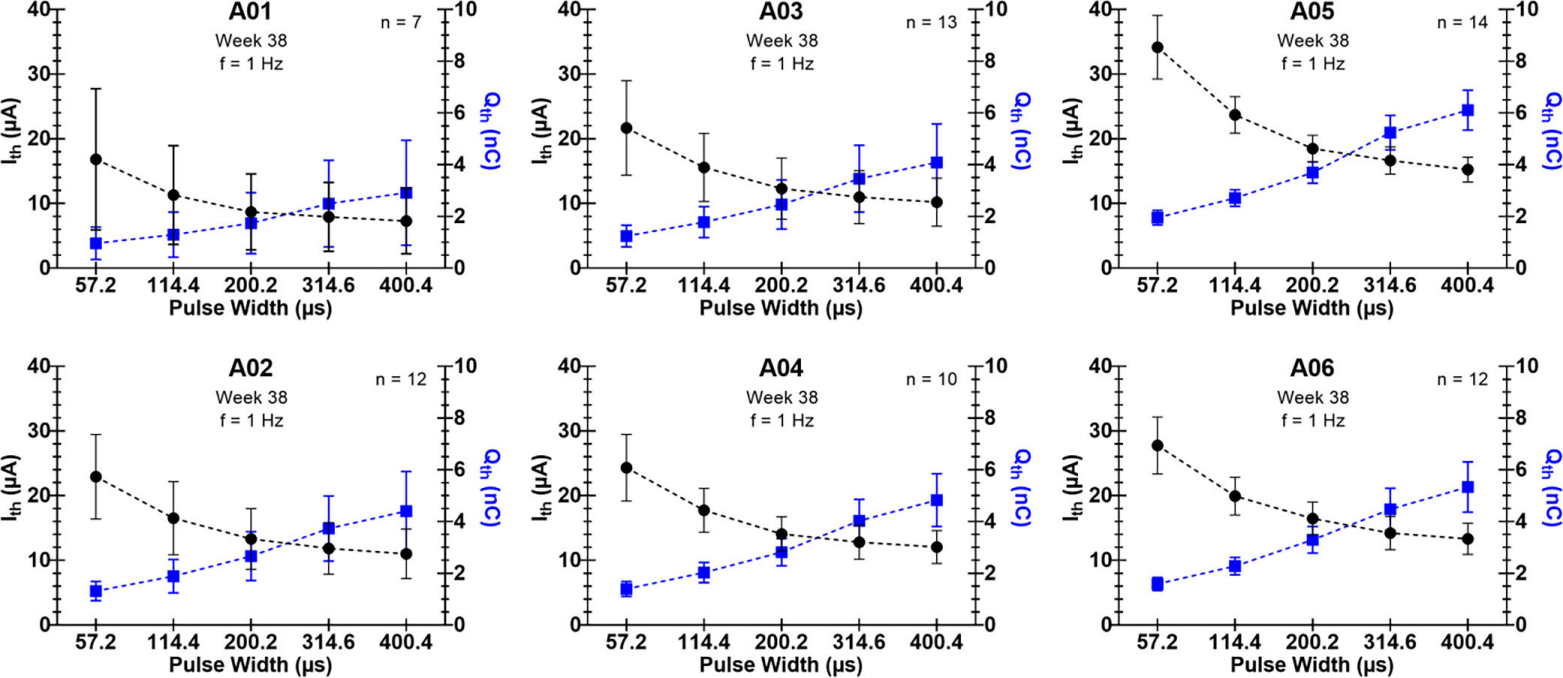
Current thresholds for motor recruitment (I_th_) as a function of pulse width (frequency set to 1 Hz) for each animal (ID listed at top of each graph). Charge values (Q_th_) were calculated from I_th_ values and are plotted in blue with values corresponding to the right y-axis. Data shows average and standard deviation for all electrodes generating movement in the hindlimb on week 38 post-implantation (n listed in top right corner of each plot).

Changing pulse width notably changed the I_th_ required to elicit movement in the limb. Increasing pulse width (at 1 Hz frequency) caused Ith to decrease for all electrodes tested. The charge delivered per pulse (at I_th_) increased with increasing pulse width. Figure 4 shows average I_th_ values as a function of pulse width. Figure 4 also shows the corresponding charge required to elicit a motor response as a function of pulse width, calculated from I_th_ values. Figure 5 shows I_th_ and Q_th_ vs pulse width for each implanted animal.

## IV. Discussion

Modulation of frequency within the 1 to 50 Hz range is an effective method for changing the type of limb movement evoked from singular twitches to sustained muscle contractions. We found the transition between these two types of movement occurred around 25 Hz, matching what was previously reported in the literature. The lack of change in I_th_ within the tested frequency range may be beneficial, as frequency modulation could be used to change limb movement without needing to increase current amplitude and supply voltage. Further work investigating frequency encoding patterns tied to specific movements is necessary to accomplish complex motor tasks in the hindlimb. Additional research is also needed to understand the impact of choice of stimulation frequency on muscle fatigue.

Stimulating with longer duration pulses required less current to elicit the same motor response. And so, pulse width modulation may be a useful tool for minimizing the current required to elicit a desired movement in the limb. This is especially helpful when using a wireless device like the WFMA, where power supply is limited to approximately 4 V. At the same time, increasing the pulse width increased the total charge delivered per pulse at the minimum current necessary for generating movement. And so, increasing pulse width may increase charge density at the electrode surface to levels beyond material limitations. Further research into the effect of pulse width modulation is needed to understand how changes in charge density may affect long-term stability of the electrode material and electrode-tissue interface.

## V. Conclusion

Effects of frequency and pulse width modulation with the WFMA were in line with previous studies of functional electrical stimulation. Intraneural stimulation at frequencies above 25 Hz caused sustained muscle contractions for the duration of applied electrical stimulation. I_th_ did not change significantly within the range of 1 to 50 Hz. I_th_ decreased with increasing pulse width between 57.2 and 400.4 μs. Results of varying stimulation parameters with the WFMA after 38 weeks of implantation in rat sciatic nerve are promising for using the device as a prosthetic for functional electrical stimulation. Future work will need to investigate control methods that best achieve desired motor tasks in the hindlimb using the wireless interface.

## Clinical Relevance—

This work establishes the efficacy of various stimulation parameters for controlling movement with a wireless peripheral nerve stimulator.
